# Does leaving an abusive partner lead to a decline in victimization?

**DOI:** 10.1186/s12889-018-5330-z

**Published:** 2018-03-27

**Authors:** Zohre Ahmadabadi, Jackob M. Najman, Gail M. Williams, Alexandra M. Clavarino, Peter d’Abbs, Nargess Saiepour

**Affiliations:** 1 School of Public Health, The University of Queensland, Herston Road, Herston, QLD 4006 Australia; 2 School of Social Sciences, The University of Queensland, St Lucia, QLD 4072 Australia; 3 School of Pharmacy, The University of Queensland, Woolloongabba, QLD 4102 Australia; 4 Menzies School of Health Research, Spring Hill, QLD 4000 Australia

**Keywords:** Intimate partner violence, Leave, Revictimization

## Abstract

**Background:**

This paper investigates gender differences in persistence of intimate partner violence (IPV), for those remaining or leaving an abusive relationship. We followed a sample of males and females to examine whether leaving an abusive partner may alter the continuity of victimization.

**Methods:**

Data were taken from the 21 and 30-year follow-ups of the Mater Hospital and University of Queensland Study of Pregnancy (MUSP) in Australia. A cohort of 1265 respondents, including 874 females and 391 males, completed a 21-item version of the Composite Abuse Scale.

**Results:**

We found proportionally similar rates of IPV victimization for males and females at both the 21 and 30 year follow-ups. Females who reported they had an abusive partner at the 21 year follow-up were more likely to subsequently change their partner than did males. Harassment and then emotional abuse appeared to have a stronger association for females leaving a partner. For males, a reported history of IPV was not significantly associated with leaving the partner. There was no significant association between leaving (or not) a previous abusive relationship and later victimization, either for male or female respondents.

**Conclusion:**

Changing a partner does not interrupt the continuity of victimization either for male or female respondents, and previous IPV victimization remained a determining factor of re-abuse, despite re-partnering.

## Background

Intimate partner violence (IPV) may occur repeatedly in the context of some intimate relationships [1]. There is some evidence to suggest that prior victimization/perpetration is a strong risk factor of further victimization/perpetration [2–5]. Although remaining in an abusive relationship is often accompanied by continued victimization, it is unclear whether leaving that relationship and re-partnering leads to a reduced risk of later victimization. Answering this question is relevant to IPV interventions that often aim to encourage the victims to leave their abusive partner, as well more general policies intended to improve outcomes for those affected by IPV [6].

Prior research following those who have previously experienced IPV has been limited and inconclusive. Only a small number of scholars have suggested that changing a partner may reduce IPV perpetration [7, 8] and victimization [9]. Capaldi et al. [7] found that those who stayed with a partner, reported higher stability in aggressive behaviors compared to those who changed partner. They suggested that IPV is “dyadic in nature” and reinforced by relationships characterized by chronic conflict. It may be that changing a partner and leaving the hostile environment associated with that partner might interrupt destructive patterns of interaction. These results, however, were based upon couples from disadvantaged and at-risk neighborhoods and the findings reported may be confounded by ecological risk factors. In another study of low income victimized women, leaving an abusive partner was found to decrease the risk of further victimization. Despite investigating a cohort of abused women, this latter study did not clarify whether these women were victimized by the partner they had left or by a new partner or both [9].

A recent body of research has cast doubt on the presumed advantages of leaving an abusive relationship. Interpreting findings from a *life course perspective* suggests that particular periods of the life course may be associated with higher rates of IPV. Characteristics of early adulthood (e.g., instability in emotions, interpersonal relationships, and career orientation) contribute to a greater risk of IPV in this period. Afterwards, developmental changes (e.g., maturing behaviors, personal achievements, interactional skills) may protect individuals from subsequent victimization/ perpetration- regardless of leaving or staying with a partner [10–13].

Another group of scholars suggest that IPV perpetration and victimization continues across relationships [3, 8, 14–18]. Leaving a prior abusive partner, might arguably increase the risk of more severe IPV victimization, particularly homicide [19]. Other than partner-related characteristics, risk of [re]victimization is partly associated with victim-related factors which may exist before the early experience of IPV. These factors include lower socio-economic resources and a past history of child abuse experienced by the victims. Re-victimization might be exacerbated across relationships because of financial hardship, poor mental health, substance abuse and having children. Relationship break-up is itself a traumatic event which may involve a long period of exposure to risk and affects further relationships [20–26].

Beside discrepancies in the literature, previous studies in the field have a number of limitations that need to be addressed: First, much of the research is restricted to female victimization [27–29]. Therefore, males’ [re]victimization as well as possible effects of leaving an abusive partner for males needs to be determined. Second, there is unresolved debate about gender differences in the consequences of leaving an abusive relationship. One group of scholars focus on the internal (e.g., psychological difficulties) and external (e.g. benefit of their children and economic dependency) barriers which impact on females’ ability to leave an abusive relationship [24, 30]. By contrast, other scholars suggest that due to multiple obstacles (e.g., expectations that males are responsible even for an abusive partner), victimized men may be unwilling to terminate their violent relationships [31, 32]. There is inadequate evidence about the extent to which different forms of IPV may predict leaving an abusive partner. Although physical and emotional abuse are mostly considered to be correlated, they might have different consequences [33]. Further, pre-existing factors may confound the association between prior and later victimization, and the consequences of leaving an abusive relationship.

This paper uses data from a long running longitudinal study to investigate whether there are gender differences in patterns of IPV continuity after leaving an abusive partner. We examine the association between different forms of IPV victimization and leaving an abusive partner. We also test whether change of partner reduces further victimization and whether there are gender differences in IPV in these repartnered relationships.

## Method

### Participants

Data for the current study were taken from the Mater Hospital and University of Queensland Study of Pregnancy (MUSP) in Australia [34], Baseline data were collected at the first antenatal visit to the Mater Public Hospital in Brisbane between 1981 and 1983 from 7223 consecutive women. Additional assessments were conducted when the study children were 6 months, 5 years, 14 years, 21 and 30 years old. The Mater Hospital and the University of Queensland Ethics committees approved this study. The present analysis uses data from the 21 and 30 year follow-up surveys. At these phases of the study, written informed consent was obtained from the participants. The sample was derived from 3271 and 2401 persons who participated in the 21 and 30-year follow-ups. A cohort of 1265 cases including 874 females and 391 males who participated at both phases and had a partner at the 21-year follow up comprised the study sample. Some 69.1% of the sample were females. The mean age at the 21-year follow up was 20.61 (SD ± 0.84) (Males = 20.61 ± 0.87; Females = 20.61 ± 0.83) and at the 30-year follow up was 30.28 (SD ± 1.12) (Males = 30.38 ± 1.17; Females = 30.23 ± 1.10). Participants’ racial background was Caucasian (94.0%), Asian (3.1%) and Aboriginal and Islander (2.9%). In the 21-year follow up 41.2% of respondents were single and were excluded from further analysis.

### Measurement

#### Intimate partner violence

We measured IPV at 21 and 30 years using a modified version of the Composite Abuse Scale (CAS) [35, 36]. The CAS is a validated and widely used measure to assess the frequency of experiences of violence in intimate relationships (in either current or previous relationships) [37, 38]. It should be noted that despite similar items, these two measures have a difference in the timing of recall of each item: at 21 years respondents were asked to recall “ever happened” incidences of IPV, but at 30 years they were asked to recall their “last year” relationships. Both questionnaires ask about respondents’ current or previous relationships.

The scale consists of same 21 items (α = 0.94 for the 21 and 0.93 for the 30 year old follow up) and 4 subscales: severe combined abuse (has two items including rape and assault with a knife or weapon; possible score 0–10), emotional abuse (α = 0.91 for 21 and α = 0.90 for 30 years; has 11 items that include keeping apart from friends and family, insults, blame and verbal violence; possible score 0–55), physical abuse (α = 0.91 for 21 and α = 0.87 for 30 years; has 4 items which include kicking, slapping, hitting; possible score 0–20) and harassment (α = 0.83 at 21 & α = 0.72 at 30 years; comprises 4 items including harassing at work and over the telephone; possible score 0–20). Response options are never (=0), only once (=1), several times (=2), once a month (=3), once a week (=4) and daily (=5) [36]. After adding the scores of each subscale, recommended cut-offs were applied (severe combined abuse (≥ 1), physical abuse (≥ 1), emotional abuse (≥ 3), and harassment (≥ 2). If a respondent’s score is equal or higher than the cut-off score, they are considered as *abused*. Finally, respondents who experienced at least one type of IPV were recoded into *abused* and those who did not report any type of IPV were categorized into *non*-*abused*.

### Changing partner at 21 years

At 30 years, length of relationship was measured with a question: *for how long (in years) has your current live-in relationship lasted* (ranged from one month to 17 years). Average length of relationship for females was 7.3 years (± 3.8) and for males was 6.3 years (± 3.6). Length of relationship was subtracted from the duration between two surveys and categorized into *those who had stayed with the same partner since the 21 year follow up* and t*hose who had changed partners after the 21 year follow up*.

### Covariates

We adjust for a number of demographic and personal variables that may be related to both early and later IPV victimization as well as leaving a relationship. Previous research suggests that [re]victimization and staying in an abusive relationship, are related to the following factors: marital status, lower socio-economic status, presence of children, history of child abuse, and poor mental health [24, 39–43].

All participants were about 21 and 30 years of age at each follow up, so we did not adjust for the age differences in the cohort. Marital status was measured by the question *what is your present marital status?* Categories comprised *single/never married, living together, married or separated*. Education levels included *high school or less, diploma and college* and *university*. Having a child was dichotomized into *yes* and *no*. Participants were asked about their family income which was defined as gross income before tax. Then using the Australian National Poverty Line as a guide [44], we categorized those respondents whose income was at or below the poverty line into *low income* and the rest into *higher income*. For measuring history of sexual child abuse 21-year respondents were asked whether they had experienced being pressured or forced to have sexual contact before they were 16 years. Depression was assessed using the Center for Epidemiologic Studies Depression Scale (CES-D) which is a widely-used self-report scale [45]. It contains 20 items measuring the current level of depressive symptoms over the past week (e.g., feeling hopelessness, restless sleep, poor appetite) in a general population (α = 0.88). Response options range from 0 (rarely/less than 1 day) to 3 most of the time/5–7 days). Scores range from 0 to 60, with high score indicating a greater level of depressive symptoms. Based on the recommended cut-off score (> 16), respondents at 21 year follow-up were grouped into two categories: non-depressed and depressed [46, 47]. Sexual orientation at 30 years was measured using a single question *during the last 12 months have your sexual partners been:* only the opposite sex, only the same sex and both sexes. We then categorised respondents into two categories of *heterosexual* and *homo/bisexual*.

### Data analysis

**Fig. 1:**
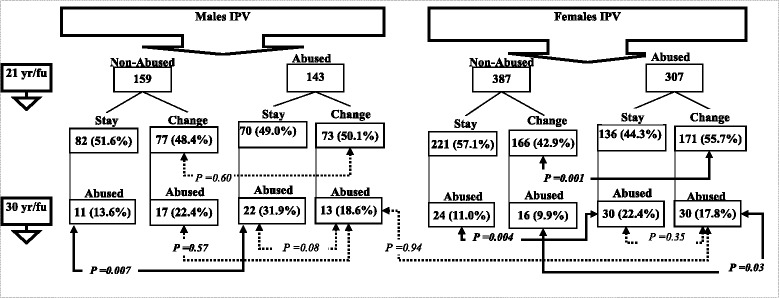
Gender differences in continuity of IPV at 21 and 30 year follow ups by staying with/changing partner. Solid arrows represent statistically significant differences between the groups (*p*-value< 0.05). Dashed arrows indicate non-significant associations (*p*-value > 0.05). *P*-values were obtained from the chi-square test

In Table 1, the prevalence of each type of IPV was determined across the gender groups, chi square and t-test were used to test the significance of differences. In Table 2, we performed a univariable logistic regression analysis for the association (expressed as the odds ratios with 95% confidence intervals) between each covariate and IPV at 30 years. In Fig. 1, we compared percentages of abused or not abused males and females, based on their choice to stay or leave, using chi square test and set a *p*-value< 0.05 for significance. In Table 3, univariable and multivariable logistic regression models were used to examine the relationship between forms of IPV at 21 years and leaving the then abusive partner, separately for males and females. Finally, an interaction term (IPV at 21 × leaving/staying) was used to examine the effect of staying/changing an abusive partner on the association between IPV victimization at 21 and 30 years. Statistical analyses were carried out using STATA-13 and SPSS-24 statistical software packages.

**Table 1:** Gender differences in study variables

	Male	Female	χ^2^(*p*-value)
	%		
Marital status at 21 yr/fu^a^	(*n* = 388)	(*n* = 872)	14.1 (< 0.001)
Living together/bf-gf	97.7	92.2	
Married	2.3	7.8	
Have Children at 21 yr/fu	(*n* = 386)	(*n* = 870)	22.4 (< 0.001)
No	95.3	86.3	
Yes	4.7	13.7	
Education level at 21 yr/fu	(*n* = 384)	(*n* = 865)	3.3 (0.19)
University	3.9	5.0	
College	21.6	25.5	
High School & less	74.5	69.5	
Employment at 21 yr/fu	(*n* = 384)	(*n* = 867)	43.7 (< 0.001)
Full time	57.3	37.7	
Part-time	28.6	37.4	
Unemployed	14.1	24.9	
Income at 21 yr/fu	(*n* = 388)	(*n* = 871)	13.3 (< 0.001)
Higher	86.1	77.2	
Low	13.9	22.8	
Depression (CES-D) at 21 yr/fu	(*n* = 386)	(*n* = 870)	11.2 (0.001)
No	85.8	77.6	
Yes	14.2	22.4	
History of sexual abuse	(*n* = 386)	(*n* = 869)	11.2 (0.001)
Non-abused	77.2	67.9	
Abused	22.8	32.1	
Sexual orientation at 30 yr/fu:	(*n* = 366)	(*n* = 822)	1.04 (0.31)
Heterosexual	94.8	96.1	
Homo/bisexual	5.2	3.9	
Change the partner of 21-year-old	(*n* = 305)	(*n* = 698)	
Yes	49.5	48.6	0.08 (0.79)
IPV victimization at 21 yr/fu^b^	(*n* = 383)	(*n* = 868)	
SC	2.9	6.5	6.7 (0.01)
PA	39.6	33.4	4.3 (0.04)
EA	27.2	31.0	1.9 (0.17)
H	17.7	22.1	3.2 (0.07)
At least one type	48.6	44.9	1.4 (0.23)
IPV victimization at 30 yr/fu^c^	(*n* = 378)	(*n* = 859)	
SC	0.8	0.8	0.01 (0.96)
PA	10.3	9.2	0.37 (0.54)
EA	16.1	16.1	0.00 (1.0)
H	7.1	6.5	0.16 (0.7)
At least one type	20.9	18.6	0.9 (0.87)
Length of current relationship at 21 yr/fu (Range: 1 month-8 years)	Mean (SD)		t-test
	1.6 (1.3)	2.0 (1.6)	4.2 (< 0.001)

^a^Respondents with no partner at 21 [single ever (*n* = 879) and single now before in relationship (*n* = 12)] were excluded from the analysis; ^b^IPV victimization at 21 years refers to life-time experiences; ^c^IPV victimization at 30 refers to last year experiences

Abbreviations *IPV* Intimate partner violence, *yr/fu* year follow-up, *SC* severe combined, *PA* physical abuse, *EA* emotional abuse, *H* harassment

**Table 2:** Univariable logistic regression analysis for study covariates and changing partner of 21-year-old and IPV at 30 year follow-up (OR; CI _95%_)

Predictors^a^	Change the partner of 21 year-old (stay = ref)		IPV victimization at 30 years^c^ (no abuse = ref)	
	Male (*N* = 306)	Female (*N* = 700)	Male (*N* = 380)	Female (*N* = 861)
Marital status at 21 yr/fu	^d^	**4.41 (2.25–8.67)**	0.92 (0.19–4.50)	0.88 (0.48–1.62)
Cohabiting (Married = ref)				
Have Children at yr/fu	^d^	**0.54 (0.34–0.84)**	**3.0 (1.16–7.74)**	1.30 (0.80–2.06)
Yes (No = ref)				
Education at 21	1.37 (0.84–2.27)	1.29 (0.94–1.78)	**0.56 (0.33–0.96)**	1.23 (0.84–1.80)
<High School (higher = ref)				
Employment at yr/fu	1.09 (0.57–2.11)	0.80 (0.56–1.14)	**2.0 (1.05–3.76)**	**1.87 (1.29–2.71)**
Unemployed (employed = ref)				
Income at yr/fu	1.51 (0.78–2.94)	0.98 (0.69–1.39)	1.12 (0.56–2.54)	1.30 (0.88–1.92)
Low (higher = ref)				
Depression at 21 yr/fu	1.45 (0.74–2.83)	**1.61 (1.11–2.33)**	**2.39 (1.28–4.46)**	**1.92 (1.32–2.80)**
Yes (No = ref)				
Childhood sexual abuse	**1.80 (1.02–3.15)**	0.98 (0.71–1.35)	1.54 (0.88–2.70)	**1.54 (1.08–2.19)**
Yes (No = ref)				
Sexual orientation (at 30 years)	**–**	**–**	1.50 (0.52–4.32)	1.34 (0.57–3.17)
homo/bisexual (heterosexual = ref)				
IPV at 21 yr/fu^b^	1.11 (0.71–1.75)	**1.67 (1.24–2.26)**	**1.73 (1.04–2.86)**	**2.06 (1.45–2.91)**
Yes (No = ref)				

Odds ratios in bold are significantly different to those of the reference category (*P* < 0.05). ^a^Each variable is modelled separately for males and females; ^b^IPV victimization at 21 years refers to at least one type IPV ever; ^c^IPV victimization at 30 refers to at least one type IPV during last year; ^d^Due to insufficient sample size, the analysis was not performed

Abbreviations *IPV* Intimate partner violence, *yr/fu* year follow-up, *OR*:odds ratio, *CI* confidence interval

**Table 3:** Univariable and multivariable logistic regression analysis for forms of IPV at 21 years predicting stay/change the partner of 21-year-old by 30 years, separately for males and females

	Males				Females			
	*n* (%)		OR (95% CI)		*n* (%)		OR (95% CI)	
	Stay(ref)	Change	Model 1^b^	Model 2^c^	Stay(ref)	Change	Model 1^b^	Model 2^c^
Individual IPV victimization at 21 yr/fu (yes = ref)^a^								
SC ^d^	3 (30.0%)	7 (70.0%)	2.43 (0.62–9.59)	–	23 (54.8%)	19 (45.2%)	0.87 (0.46–1.62)	0.70 (0.36–1.38)
PA	56 (49.1%)	58 (50.9%)	1.09 (0.69–1.74)	1.23 (0.73–2.09)	110 (44.9%)	124 (55.1%)	**1.47 (1.07–2.02)**	**1.54 (1.08–2.20)**
EA	38 (49.4%)	39 (50.6%)	1.06 (0.63–1.79)	0.93 (0.52–1.65)	88 (42.3%)	120 (57.7%)	**1.68 (1.21–2.34)**	**1.66 (1.16–2.38)**
H	25 (47.2%)	28 (52.8%)	1.18 (0.65–2.13)	0.95 (0.49–1.84)	52 (35.6%)	94 (64.4%)	**2.26 (1.55–3.30)**	**2.12 (1.40–3.20)**
At least one type	70 (49.0%)	73 (51.0%)	1.11 (0.71–1.75)	1.25 (0.76–2.06)	136 (44.3%)	171 (55.7%)	**1.67 (1.24–2.26)**	**1.70 (1.22–2.36)**
Multiple IPV victimization at 21 yr/fu								
None (ref)	82 (51.6%)	77 (52.1%)	1	1	221(57.1%)	166 (42.9%)	1	1
One type	34 (47.9%)	37 (52.1%)	1.16 (0.66–2.03)	1.67 (0.89–2.15)	56 (50.0%)	56 (50.0%)	1.33 (0.87–2.03)	1.35 (0.86–2.11)
Two and more	36 (50.0%)	36 (50.0%)	1.07 (0.61–1.86)	0.94 (0.50–1.75)	80 (41.0%)	115 (59.0%)	**1.91 (1.35–2.71)**	**1.97 (1.34–2.92)**

Odds ratios in bold are significantly different to those of the reference category (*P* < 0.05). ^a^Each form of IPV is modelled separately for males and females. ^b^Unadjusted odds ratios; ^c^Adjusted for marital status, having children, education, employment, income, history of childhood sexual abuse, and depression, all measured at 21 years. In this model, IPV forms, are not mutually exclusive

^d^Due to males’ insufficient sample size in SC, multivariate regression analysis was not conducted

Abbreviations *IPV* Intimate partner violence, *yr/fu* year follow-up, *SC* severe combined, *PA* physical abuse, *EA* emotional abuse, *H* harassment

## Results

Among 1265 21-year-old participants who were in a relationship, 6.1% were married and 93.9% were living together. 11.1% of respondents had children.

Table 1 presents comparative information for males and females. At 21 years females are more likely to have children, to be unemployed, depressed, and with a low income. Females also report higher rates of past childhood sexual abuse. At 21 years, severe combined victimization and harassment (borderline significance; *p* = 0.07) is experienced more often by females. By contrast, 21-year-old males more frequent report being physically abused. At 30, there are no gender differences in any form IPV.

Table 2 presents the univariable associations between covariates and changing partner of 21-year-old and also IPV victimization at the 30 year follow-up for males and females. Depression and unemployment at 21 years are significant predictors of IPV at 30 years in both males and females. Males with children and females with a history of child abuse more often report the experience of IPV at 30 years.

Table 2 also shows that except for a past history of sexual child abuse, none of study variables are statistically associated with changing a partner for males. Females who cohabit, who have depression and have no child are more likely to have changed partner by the 30-year follow up.

A further detailed analysis (data not shown) suggests that although the association between having children and females’ leaving their partners is negative (Table 2), when mothers experience emotional abuse (having children × EA), the odds of changing partner increases [OR = 2.97 (CI95% = 1.13–7.83)]. We also found that while there is no significant association between females’ low income and leaving a partner [OR = 1.0 (CI95% = 0.62–1.58)], low income women who experience physical abuse (low income × PA), are significantly less likely to leave the abusive partner [OR = 0.43 (CI95% = 0.20–0.94)].

Table 3 presents the univariable and multivariable associations between forms of IPV and changing a partner. Model 1 (unadjusted odds ratio) and Model 2 (adjusted for demographic variables) suggest that with the exception of severe combined abuse, females who experience physical abuse, emotional abuse and harassment at 21 years are more likely to change their partners. For males, there is no statistically significant association between the IPV and leaving their partner. The results remain significant after adjusting for the study variables (Table 3).

Figure 1, provides a flow diagram of the pattern of victimization-revictimization by change of partner for males and females. For males, we note that the percentage who changed partners between the 21 and 30 year follow-ups was similar, irrespective of whether they met the criteria for experiencing IPV. For males who had reported IPV at 21 years and changed partners, 18.6% reported experiencing IPV at the 30 year follow up, a proportion below the 31.9% of abused males at 21 who remained with their partners (borderline significant, *p* = 0.08). For females we note that 55.7% of those experiencing IPV changed partners, while 42.9% of those not experiencing IPV changed partners (*p* <  0.001) by the 30 year follow up. Females who reported IPV at 21 years and remained with their partners, were no more likely to be abused at 30 years compared to females who had changed partners (22.4% vs. 17.8%, *p* = 0.35). A further interaction terms between the experience of IPV at 21 (non-abused/abused) as *primary variable* and change of partner (stay/change) as *moderator* was conducted to predict the experience of IPV at 30 (non-abused/abused) separately for females and males (data not shown in a table). Consistent with the findings in Fig. 1, this analysis showed no association between leaving an abusive relationship and later IPV victimization, neither for females nor for males. For females, there was no significant difference in experiencing IPV at 30 years between abused females who changed their abusive partners and abused females who stayed (OR = 0.77, CI_95_ = 0.44–1.35). In contrast, the primary effect of experiencing IPV at 21 remains a robust significant predictor for experiencing IPV at 30 years. For males, no statistically significant difference in experiencing IPV at 30 years is observed between males who left their abusive or non-abusive partners (OR = 0.49, CI_95_ = 0.23–1.09). These findings were independent of a range of potential confounding factors.

## Discussion

The current study has compared males and females in the continuity of IPV victimization at 21 and 30 years of age. A cohort of 1260 cases was followed to determine whether early IPV victimization was associated with leaving the prior partner and subsequent IPV. In addition, we followed both males and females to examine how a change of abusive partners may alter the continuity of victimization.

The results of this study suggest that rates of IPV victimization declined from 21 to the 30 year follow up (41.1% vs. 20.1%; *p* <  0.0001). Despite this decline in the IPV rate across time, there was a robust significant association between early victimization and re-victimization for both males and females. We also found that a substantial proportion of females (55.7%) and males (51.0%) who report experiencing IPV at 21 years left their partners (*p* > 0.05). Victimized males at 21 years were no more likely to change partners, than those not experiencing IPV at 21 years. These findings were not affected by any of the sociodemographic factors that were considered. Harassment and then emotional abuse appeared to have a higher association with leaving partner in females. Relationship change did not appear to prevent males and females from the continued experience of victimization. We found experiencing IPV at 21 remains a robust significant predictor for experiencing IPV at 30 years, regardless of whether there is a change of partner.

The observed decline in IPV victimization from 21 to 30 years may reflect the longer period in which 21-year respondents were asked about their experiences, compared to that of 30 years (last year). Nevertheless, this finding is consistent with a *life course perspective* which suggests higher rates of IPV victimization/perpetration in emerging adulthood (ages 18–25). Features of this life course stage include instability (in emotions, living residence and career) and tendency to postpone adults’ responsibilities (e.g., commitment and parenting), which may contribute to a higher rate of IPV victimization at this period [10, 12, 13]. Transition to adulthood is associated with long-term commitments, family formation, pro-social networks, employment, developing an independent personal identity and less risk-taking and anti-social behaviors [10].

A relationship between early and further victimization supports previous research which suggests that earlier victimization may be taken to mean violence is considered a normal aspect of intimate relationships. Prior experiences of family violence may lead to cumulative disadvantages (mentally and socially) which negatively affect the nature of future relationships [4, 12, 48–51].

Slightly higher rates of leaving the abusive partner in females seems to be consistent with other research indicating that females have disproportionately higher rates of relationship termination than males [32]. Considering different forms of IPV, the experience of harassment and then emotional abuse had a stronger association with leaving an abusive partner. This may be explained by females’ emotion-focused preferences and expectations from an intimate relationship [52]. Being in a relationship, characterised by harassment, controlling behaviours and hurting feelings threatens females’ well-being, possibly more than the experience of physical abuse [33, 53]. Consequently, females may more often decide not to remain in such a relationship.

We also found no support for the effectiveness of leaving an abusive partner. This finding is in line some previous studies which find no significant difference between those who stayed or changed partners [14, 15]. However, our results differ from those of Short, et al. [8] which showed that re-partnering with a less aggressive woman disrupts males’ psychological and physical aggressive behaviors.

A body of research has shown that earlier victimization may lead to long-lasting consequences for survivors like fear, posttraumatic stress, anxiety and disempowerment [54]. Survivors may carry negative outcomes of earlier victimization to the future relationships. Further research might explore mediators and moderators between early and later IPV experiences. A third explanation for these findings might focus on socio-cultural factors, including acceptance of violence and gender role norms, which remain across relationships [29]. It may be the case that leaving a violent partner may not necessarily mean leaving the structural context of intimate partner violence. Lack of available social support for those who leave their abusive relationships may explain the ineffectiveness of leaving.

## Conclusions

This study has several strengths: IPV was assessed by a validated measure at 21 and 30 years. We also used the longitudinal data from a large prospective cohort of both males and females and adjusted for a range of confounding factors. We have found that leaving an abusive relationship makes no significant difference to experiencing further IPV and early IPV victimization remains the strongest predictor of re-abuse, despite changing partner. The current work has extended existing knowledge about IPV victimization experienced in relationships with different partners. Our findings raise the question of whether there are characteristics of those affected by IPV and socio-cultural factors, not measured in this study, that need to be identified and addressed if IPV and its consequences are to be reduced.

The findings of this study have significant implications for IPV reduction programs: first, gender differences in predictors of IPV and in its association with leaving a partner raise the need for *gender specific IPV interventions*. For example, we found similar rates of IPV for 30-year-old males and females and no association between the experience of IPV and males’ leaving their partner. These findings leads to a recommendation that gender-specific prevention efforts should put greater emphasis on males’ IPV victimization and their decision to stay in an abusive relationship.

The finding that early IPV victimization remained a determining factor of revictimization highlights the need for *early IPV prevention*. If it is possible to prevent first victimization experiences, then the subsequent victimization may be avoided [8]. More importantly and before any intimate relationship and violence occurs, comprehensive primary prevention should address protective/risk factors of IPV. Continued efforts are needed to prevent childhood sexual abuse as an important risk factor for IPV in adulthood. We found that having a child at a young age (21 years old) was a strong risk factor for further males’ victimization. This finding can be used to develop targeted interventions aimed at early fatherhood. Care of children exposed to IPV and their health and well-being should be acknowledged in IPV interventions.

IPV interventions which protect and assist those affected by IPV, should address complex needs of survivors. For example, in the current study, depression was a significant predictor of both changing partner and IPV victimization. Clinical intervention efforts are required to target pre-existing as well as subsequent mental health problems of victims to minimize the risk of further abuse. Having a low income was also a significant barrier against abused females to leave an abusive partner. IPV interventions should therefore consider the policy of women’s financial empowerment [21].

This study has a number of limitations: The data were collected using a self-report measure from one partner, which may associated with self-serving bias or over-reporting negative behaviours of partners. In addition, at 21 years respondents reported their life-time IPV in either their current or previous relationships, while at the 30 year follow up, they described their most recent relationships during last 12 months [35]. Males’ lower sample size may decrease statistical power to detect differences for males. Another issue is that MUSP used a population sample which might not include those who have experienced very severe levels of intimate partner violence [55]. The possibility of endogeneity has not been addressed in the study. There are a number of confounders that have not been considered (given the sample size there was a limit to the variables included in the final model). Further some of the possible cause-effect associations could not be tested, for example while the study has very detailed data on early life course aggression, introducing this detail would require a different paper. Given that the key research question is whether there is a reduction in IPV in the affected person leaves their partner, the findings are consistent and unlikely to change with the introduction of additional variables.

## Abbreviations

CAS: Composite Abuse Scale
CES-D: The Center for Epidemiologic Studies Depression Scale
CI: Confidence Interval
EA: Emotional Abuse
H: Harassment
IPV: Intimate Partner Violence
MUSP: the Mater Hospital and University of Queensland Study of Pregnancy
OR: Odds Ratio
PA: Physical Abuse
SC: Severe Combined
